# Using the ClinFIT COVID-19 Instrument to Assess the Functional Impairments Specific to Post-COVID-19 Patients in Romania

**DOI:** 10.3390/diagnostics14141540

**Published:** 2024-07-17

**Authors:** Clara Ursescu, Gigi Teodoru, Sandica Bucurica, Remus Iulian Nica, Ștefan Dragoș Lazăr, Marius Nicolae Popescu, Ileana Ciobanu, Mihai Berteanu

**Affiliations:** 1 Department of Rehabilitation Medicine, Carol Davila University of Medicine and Pharmacy, 020021 Bucharest, Romania; clara-catalina.ursescu@drd.umfcd.ro; 2 Department of Rehabilitation Medicine, University Emergency Central Military Hospital “Dr. Carol Davila”, 010825 Bucharest, Romania; 3 Department of Internal Medicine and Gastroenterology, Carol Davila University of Medicine and Pharmacy, 020021 Bucharest, Romania; 4 Department of Gastroenterology, University Emergency Central Military Hospital “Dr. Carol Davila”, 010825 Bucharest, Romania; 5 Discipline of General Surgery, Faculty of Midwifery and Nursing, Carol Davila University of Medicine and Pharmacy, 050474 Bucharest, Romania; 6 Department of General Surgery, University Emergency Central Military Hospital “Dr. Carol Davila”, 010825 Bucharest, Romania; 7 Department of Infectious Diseases, Carol Davila University of Medicine and Pharmacy, 020021 Bucharest, Romania; dragos.lazar@umfcd.ro; 8 Department of Physical and Rehabilitation Medicine, Carol Davila University of Medicine and Pharmacy, 020021 Bucharest, Romania; marius.popescu@umfcd.ro (M.N.P.); ileana.ciobanu@umfcd.ro (I.C.); mberteanu@gmail.com (M.B.)

**Keywords:** post COVID-19 rehabilitation, post COVID-19 sequelae, ClinFIT COVID-19, functional domains, ICF

## Abstract

Introduction: The COVID-19 pandemic has led to approximately 3.5 million cases in Romania, causing systemic inflammation and over 200 symptoms affecting various body systems. This complexity has challenged rehabilitation systems, necessitating personalized plans tailored to each patient’s illness stage and impairment level. The ISPRM-developed ClinFIT COVID-19 instrument, aligned with the ICF categories, assists in assessing patients during acute, post-acute, and long-term phases. Objective: This study aimed to evaluate and assess functional impairments in post-COVID-19 patients in Romania, with a secondary goal of generating rehabilitation directions. Methods: Data were collected from patients at two Bucharest medical centers, including those with persistent symptoms post-acute phase. Participants were assessed using the adapted ClinFIT COVID-19 instrument, and descriptive statistics were applied. Conclusions: Findings revealed diverse functional impairments in physical, psychological, and social domains among post-COVID-19 patients, with severe impairments more common in those with long-term COVID-19. Complete impairment in complex movement and paid work was noted, affecting one-third of salaried employees and forcing some to retire. In the acute phase, the most frequent functional impairments were sleep, attention, pain sensation, and exercise tolerance functions. In contrast, the most severely affected functions were exercise tolerance and mobility joint functions. Age did not positively correlate with any of the analyzed functions. In the post-acute phase, sleep, energy, and drive functions remained the most frequently affected functions, while the most severely affected was, by far, the moving around function. In the post-acute period, respiratory and respiratory muscle functions strongly correlated with all tasks related to physical activity. In the long COVID-19 phase, remunerative employment was the most severely affected function, while attention functions remained the most frequently affected, similar to the acute phase. The ClinFIT COVID-19 instrument effectively captured these impairments, underscoring the need for comprehensive rehabilitation strategies.

## 1. Introduction

The Coronavirus Disease 2019 (COVID-19) pandemic, which emerged in December 2019 in Wuhan, was declared a public health emergency of international concern by the World Health Organization on 30 January 2020, due to infection with severe acute respiratory syndrome—coronavirus 2 (SARS-CoV-2) [1,2]. Since then, there have been over 770 million cases of infection globally, including approximately 3.5 million cases in Romania [2]. According to the definition, a person was considered positive if they had a positive nucleic acid amplification test, regardless of whether they were symptomatic, or if they met clinical and/or epidemiological criteria and had a positive SARS-CoV-2 antigen test for professional or self-testing purposes [3]. The systemic inflammation caused by COVID-19 has led to the exacerbation of pre-existing lesions and the emergence of new dysfunctions in various systems and organs [4]. Over 200 symptoms have been identified, impacting multiple structures and functions of the body, such as cardiovascular, thrombotic, and cerebrovascular diseases, diabetes mellitus, myalgic encephalomyelitis and chronic fatigue syndrome, and autonomic system impairments such as postural orthostatic tachycardia syndrome [5].

Given the variety of symptoms induced by COVID-19, expanding the rehabilitation system to assess the functional limitations faced by patients with or post-COVID-19 has been a significant challenge. The assessment aimed to generate a personalized rehabilitation program and, more importantly, to guide healthcare professionals in optimally responding to patients’ medical care needs [6].

For these patients, the need for rehabilitation varies depending on the phase they are in (acute, post-acute, or late) and the type and severity degree of impairment. UEMS-PRM recommendations have been towards the implementation of an individualized rehabilitation plan tailored to the patient’s age, medical condition, previous functioning, comorbidities and current complications, activity limitations, and participation restrictions as influenced by personal and environmental conditions [7]. To address this issue, the International Society of Physical Medicine and Rehabilitation (ISPRM) has developed an instrument for assessing and reporting the functioning of patients with COVID-19—“ClinFIT COVID-19”. This instrument is based on the International Classification of Functioning, Disability, and Health (ICF) categories, for which ClinFIT COVID-19 finds correspondence in acute, post-acute, and long-term states [6]. The assessment and quantification of disability were based, until the emergence of COVID-19, on the International Classification of Functioning, Disability, and Health (ICF), an instrument developed by the World Health Organization (WHO) in 2001, which aimed to describe and establish a common method of expression and communication, for evaluating health status and related issues. In addition to the ICF, in 2010, the WHO developed a new tool for assessing the degree of disability that would allow for the determination of a single functioning score from evaluations of specific activities, known as the WHO Disability Assessment Schedule (WHODAS 2.0) [8]. This was followed by the Functional Independence Measure (FIM) score, clinical instruments such as the ICF Generic, Rehabilitation, and Core Sets for characterizing functioning and disability, and “simple, intuitive descriptions” contained in the ICF Generic and Rehabilitation Set [9].

Our research had two objectives. The primary objective was to evaluate and assess the characteristics of functional impairment in post-COVID-19 patients in Romania.

As a secondary endpoint, we aimed to generate rehabilitation directions for restoring the functioning of patients facing COVID-19 and post-COVID-19 conditions.

## 2. Materials and Methods

The present study was part of a larger, international, multicentric, cross-sectional study in which Romania actively collaborated by collecting data under the auspices of ISPRM from April 2020 to July 2023.

Only the data resulting from patients in Romania were presented. The patient collection centers were the Central Military Emergency University Hospital Dr. Carol Davila, Bucharest—Department 2 of Neurological Rehabilitation, The COVID-19 Diagnostic Medical Center from University Emergency Central Military Hospital “Dr. Carol Davila”, Bucharest, Romania, and the Clinic Department II of Adult Infectious Diseases from Clinical Hospital of Infectious and Tropical Diseases, Bucharest.

Inclusion criteria: adults (>18 years) with ongoing signs and symptoms of COVID-19 at <12 weeks from disease onset or patients with post-COVID-19 condition (individuals experiencing signs and symptoms compatible with COVID-19 that were not explained by an alternative diagnosis and continued for more than 12 weeks after onset) and could understand the study’s purpose and sign the informed consent form.

The terms were defined according to the World Health Statistics 2021, National Institute for Health and Care Excellence (NICE) COVID-19 rapid guideline, and the World Health Organization’s case definition of the post-COVID-19 condition by a Delphi consensus [1,2,3].

Therefore, the COVID-19 acute context was defined by those persons experiencing signs and symptoms for up to 4 weeks after onset, and the post-acute context was characterized by persons who continue to live with COVID-19, with ongoing signs and symptoms of COVID-19 from 4 to 12 weeks after onset. Accordingly, the long-term COVID-19 status included those persons with post-COVID-19 conditions, living with signs and symptoms consistent with COVID-19 that were not explained by an alternative diagnosis and which continued for more than 12 weeks after onset.

Exclusion criteria: Patients who were not independent in performing daily activities before the onset of COVID-19 or patients with neurological, orthopedic, or cardiopulmonary diseases before the onset of COVID-19.

Data collection included time, days from onset, required hospitalization, need for oxygen/ventilation, need for assistance from others or devices, type of rehabilitation services provided by the rehabilitation service provider, and the following socio-demographic data: age, gender, living situation, and employment status.

Data collection instrument: The acute version of ClinFIT COVID-19 has 13 items, the post-acute version has 15 items, and the long-term version has 16 items. For each of these versions, there are three scoring options: the 0–4 scale, the 0–10 numeric rating scale, and the 0–4 rating scale with specifications.

Data analysis/synthesis: Descriptive statistics were used to describe the study population and response distributions.

Patients were recruited at any time during the rehabilitation process. Participants were recruited by the local study coordinator, respective admission staff, and clinical personnel (medical, nursing, and therapy). The recruitment staff received a summary outlining the study objective and inclusion criteria. When recruitment was initiated by the admission staff or by the COVID-19 Diagnostic Medical Center of C.M.E.U.H. coordinator, that person informed the local study coordinator about the potential participant. The study coordinator then reconfirmed the patient’s suitability based on the inclusion criteria, obtained informed consent from the patient, and administered ClinFIT COVID-19. The study was approved by the Ethics Committee of the two participating units, the Central Military Emergency University Hospital and the Clinical Hospital of Infectious and Tropical Diseases (578/15 February 2023, 2638/20 February 2023).

Participants received a written presentation of the study and a verbal explanation of the study information. After verbal agreement to participate, the patient was asked to read and sign the informed consent form. Each patient with consent was assigned a unique participant identification number. The informed consent form was available in Romanian. No participants with physical impairment or cognitive problems were prevented from signing the consent form due to such limitations.

From the options provided by the former ISPRM ClinFIT Task Force, for the actual ClinFIT Committee, we used the ClinFIT COVID-19 version with intuitive scaling from 0 to 4 for acute and post-acute or long COVID-19 conditions [10]. Each response option to the assessment was as follows: 0: No problem; 1: Mild problem; 2: Moderate problem; 3: Severe problem; and 4: Complete problem. Each item was evaluated from 0 to 4, considering the clinical significance of the item following the corresponding simple description and the results of routine clinical instruments (e.g., physical examination, medical history, clinical tests, questionnaires). We used the ClinFIT COVID-19 Manual version 2, according to which a mild problem includes a problem that does not affect the patient’s daily activities, moderate problems may consist of a problem exceeding 1 but remaining relatively minor (<50%), a severe problem implies significant impairment greater than or equal to 50%, and a complete problem means total function impairment and is defined for each category separately.

**Figure 1:**
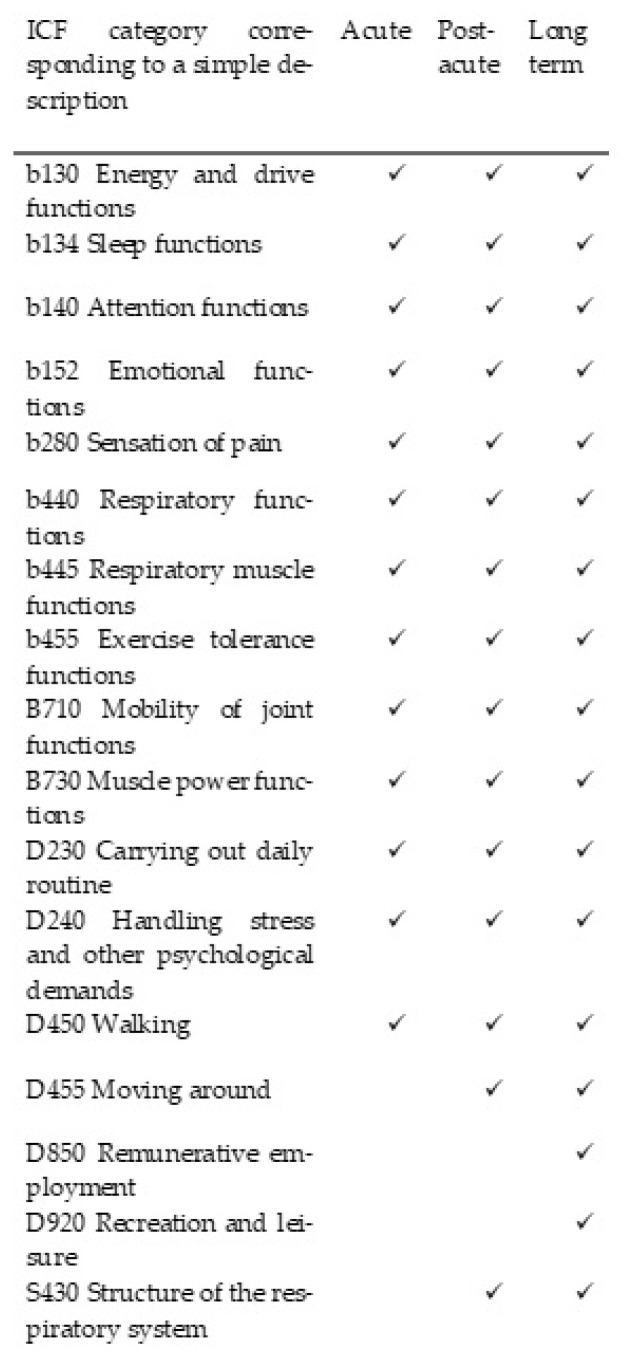
ICF categories corresponding to simple descriptions, distributed for each phase of COVID-19.

According to the protocol criteria, patients facing signs and symptoms for up to 4 weeks from onset were categorized as acute, those with continuous signs and symptoms of COVID-19 from 4 to 12 weeks after onset were categorized as post-acute, and those withing the long-term post-COVID-19 context were individuals experiencing signs and symptoms compatible with COVID-19 that were not explained by an alternative diagnosis and continued for more than 12 weeks from onset. The evaluation was performed by a physical medicine and rehabilitation specialist, respecting the ICF category descriptions and the ClinFIT COVID-19 Assessment Manual (specification assessment option) (Figure 1).

According to the definition of vaccination status, patients with three doses of vaccine were considered fully vaccinated, and patients with one or two doses were considered incompletely vaccinated. Persons who did not receive any vaccine were considered unvaccinated [11].

### Statistical Analysis

The indicators used in analyzing the patients are assessed on an impairment scale from 0 to 4, where 0 represents “Unaffected” and 4 means “Complete”. The data are presented both overall and by COVID-19 categories. Pearson correlations are used to determine the relationship or association between two variables. This is based on the covariance method and is one of the best methods for measuring variables of interest. For all Pearson correlations, a significance level of 0.01 is used. Correlations are divided into 3 categories: strong, above 0.700; moderate, between 0.300 and 0.700; and weak, below 0.300. The data are processed with IBM SPSS Statistics 25.0 (IBM Spss Statistics).

## 3. Results

### 3.1. Demographic, General, and Specific Functional Domain Data

The study participants consisted of 49 patients with COVID-19, of whom 23 were females (47%) and 26 were males (53%). Their ages ranged from 19 to 75, with a mean age of 52.59. The 49 participants were divided as follows: 18 acute (37%), 16 post-acute (33%), 15 long (31%). Patients came from 74% urban and 26% rural areas; 60% were employed, 31% were retirees, and 10% were retired due to COVID-19. The data were analyzed regarding SARS-CoV-2 vaccination status, the need for oxygen administration and assistance, and multiple functional domains. The specific functional domains included in the ClinFIT tool were energy and drive functions, sleep, attention, emotional functions, the sensation of pain, respiratory and respiratory muscle functions, walking, moving around, carrying out daily routines, and handling stress. These were in addition to exercise tolerance, mobility of joints, and muscle power functions. Also, remunerative employment state, recreation, and leisure areas of life were assessed. One of the evaluated items that not included in the functional category was the structure of the respiratory system, which is involved in COVID-19 pathology.

Most of the analyzed patients did not require oxygen administration nor assistance from another person, and the percentage of those who did was relatively equal in all phases, with a slightly higher rate in the acute phase. Although the highest percentage of patients did not require assistive devices, most of the patients who needed these were found in the post-acute phase.

Overall, most of the analyzed patients’ functions were mildly or moderately affected. The frequency of severe impairment was higher in patients in the long COVID-19 phase. Complete function impairment was encountered only in the long COVID-19 phase for complex movement and paid work.

The energy and drive function predominantly presented moderate impairment in all three COVID-19 phases, being moderately affected in a slightly higher proportion in the long COVID-19 phase.

**Figure 2:**
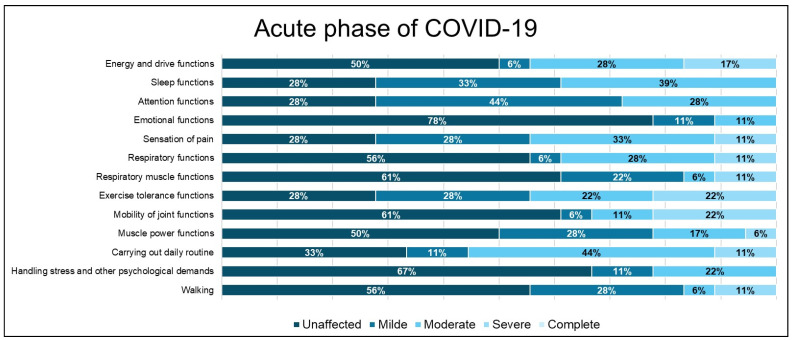
Distribution of functional domain impairment in the acute COVID-19 phase.

**Figure 3:**
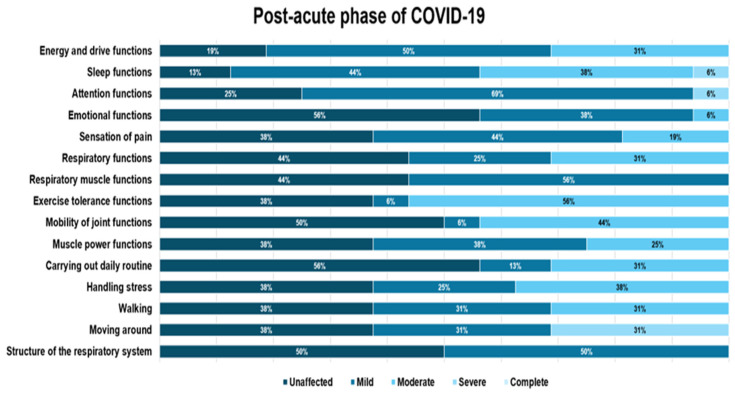
Distribution of functional domain impairment in the post-acute COVID-19 phase.

**Figure 4:**
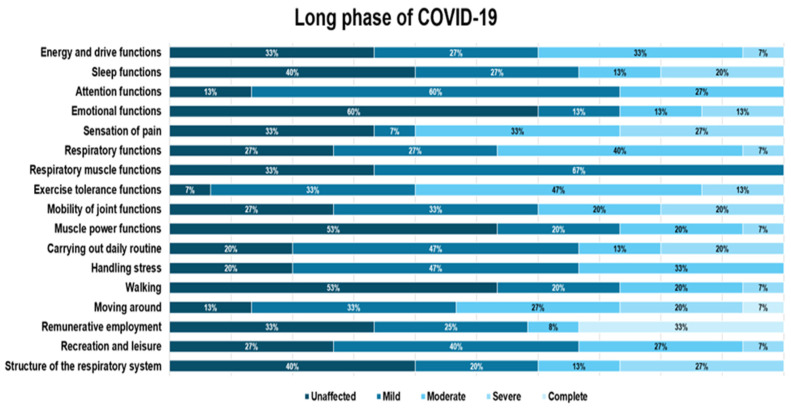
Distribution of functional domain impairment in the long COVID-19 phase.

Overall, sleep function had a higher proportion of mild impairment, with the highest percentage found in the post-acute phase, at 43.8%. Attention functions were most affected in the post-acute phase, with mild impairment, similar in percentage to the impairment in the long COVID-19 phase. Emotional functions were mildly affected in most cases, with most individuals affected in the post-acute, acute, and long COVID-19 phases, showing similar impairment. Overall, emotional functions were affected in a relatively low percentage: 34.7% (Figure 2, Figure 3 and Figure 4).

Regarding pain sensation function impairment, this was moderate and had a balanced distribution of impairment levels in the acute and long COVID-19 phases (33.3%) compared to the group of patients in the post-acute phase, where the impairment was predominantly mild (Figure 2 and Figure 4).

Respiratory function impairment was most prevalent to a moderate degree in all three phases, but the highest percentage of severe impairment was encountered in the acute phase. Exercise tolerance functions were affected overall by a significant 74.5%, with the highest proportion being moderate impairment at 40.8%. The highest percentage with moderate impairment of effort tolerance function was encountered in the post-acute phase (56.3%), but most patients without impairment of effort tolerance function were also found in this category (Figure 2 and Figure 3).

Muscle power functions were affected overall to a greater degree in the mild impairment category, but in the long COVID-19 phase, the proportion of mild impairment was equal to that of moderate impairment (Figure 4).

Overall, most patients showed mild impairment in their usual daily routine, with a higher proportion of moderate impairment in the acute phase. In the long COVID-19 phase, the predominant impairment level in carrying out daily routines was mild, different from work duties.

Handling stress and other psychological demands was one of the functions that presented a predominantly mild and moderate impact, with the highest percentage of patients with impairment found in the long COVID-19 phase (mild impairment in proportion to 46.7%), followed by moderate impairment in the post-acute phase (37.5%).

Walking was one of the functions that was mildly or severely impaired, while the other analyzed parameters were mostly mildly affected.

Moving around was one of the functions evaluated only in the post-acute and long phase of COVID-19, usually when the patient returns to his environment, and was found affected in 62% of patients in the pos-acute phase and 87% of those in the long phase of COVID-19. It was also the function that presented the most severe impairment of all those analyzed in the post-acute phase (31.3%).

Remunerative employment was completely affected in one-third of the patients who were salaried employees. These patients retired after the damage generated by COVID-19, resulting in a condition that was not compatible with the type of activity performed. Of the total analyzed patients, 80% were salaried at the time of the COVID-19 infection. The percentage was calculated only for salaried patients. Recreation and leisure were slightly affected, mostly for the group of individuals who faced the long COVID-19 stage of the infection. The structure of the respiratory system was slightly affected in a proportion equal to that of unaffected patients, while in the chronic phase, the proportion of patients with severe damage to the respiratory system was predominant (26%).

**Figure 5:**
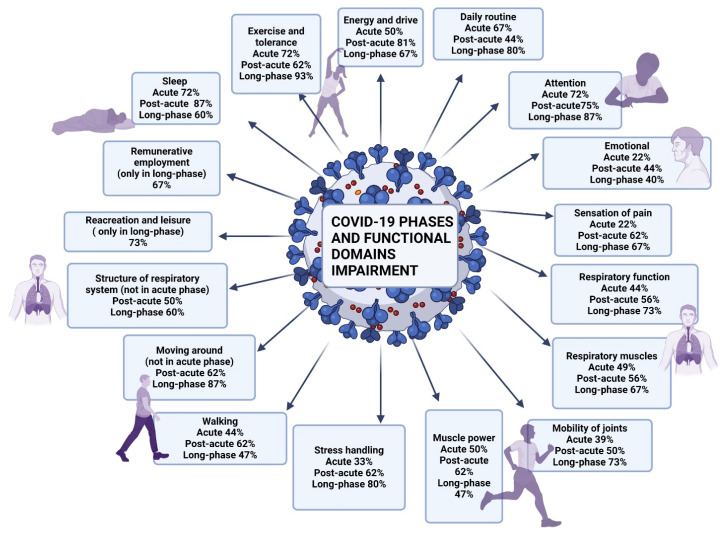
Functional domain impairment in all COVID-19 phases.

Overall, the most prevalent impaired function in all COVID-19 phases was attention, followed in similar proportion by exercise and recreation functions as well as sleep functions (Figure 5).

### 3.2. Correlation of Functional Impairments in Acute COVID-19 Patients

In the acute phase of COVID-19, energy and impulse functions were closely correlated with sleep functions, daily routine achievement, and coping with stress and other psychological manifestations (Table 1).

Regarding the need for oxygen, a solid correlation was statistically demonstrated mainly with respiratory functions but also firmly with joint mobility functions, walking, respiratory muscle functions, and effort tolerance (Table 2).

The need for assistance from another person strongly correlated with respiratory functions, joint mobility functions, and walking in the acute phase (Table 2).

The energy and drive functions, attention, emotional functions, handling stress, and other psychological demands are negatively correlated with age in the acute phase of SARS-CoV-2 infection (Table 2).

### 3.3. Correlation of Functional Impairments in Post-Acute COVID-19 Patients

Regarding the need of oxygen, assistance, and assistive devices, they were strongly correlated with carrying out daily routines followed by walking and respiratory functions. A negative correlation was found between patients’ age and attention functions (Table 3).

In the post-acute period, a significant correlation was demonstrated between respiratory functions, respiratory muscle functions, and all functions related to physical activity, with a *p*-value of 0.000. In this phase, the strongest relationship was between walking and moving around, with the walking function being included in the functional domain of moving around (Table 4).

### 3.4. Correlation of Functional Impairments in Long COVID-19 Patients

In the long COVID-19 phase, the close correlation between energy and impulse functions, sleep functions, and daily routine achievement reappeared, similarly to the acute phase (Table 1 and Table 5). A moderate correlation can be noticed between sleep functions, emotional functions, and leisure time. Mental function in the long COVID-19 phase is analyzed in the temporal context of prolonged symptom persistence and chronic exhaustion of psychological resources (Table 5).

**Table 4:** Impaired function correlations in the post-acute COVID-19 phase.

	Pearson Correlation Coefficient in Functional Domain Impairment in Post-Acute COVID-19 Phase														
FD	b130	b134	b140	b152	b280	b440	b445	b455	b710	b730	d230	d240	d450	d455	s430
b130	-	0.719 **	0.290	0.587 *	0.046	0.550 *	0.520 *	0.626 **	0.290	0.604 *	0.648 **	0.622 *	0.665 **	0.611 *	0.359
b134	0.719 **	-	0.661 **	0.523 *	0.124	0.444	0.585 *	0.664 **	0.280	0.487	0.400	0.555 *	0.520 *	0.416	0.320
b140	0.290	0.661 **	-	0.733 **	−0.046	0.393	0.566 *	0.508 *	0.174	0.316	0.249	0.622 *	0.312	0.251	0.180
b152	0.587 *	0.523 *	0.733 **	-	−0.211	0.595 *	0.514 *	0.483	0.158	0.523 *	0.679 **	0.825 **	.555 *	0.572 *	0.204
b280	0.046	0.124	−0.046	−0.211	-	0.465	0.640 **	0.595 *	0.785 **	0.400	0.024	0.298	0.397	0.327	0.775 **
b440	0.550 *	0.444	0.393	0.595 *	0.465	-	0.900 **	0.873 **	0.821 **	0.911 **	0.850 **	0.926 **	0.959 **	0.963 **	0.875 **
b445	0.520 *	0.585 *	0.566 *	0.514 *	0.640 **	0.900 **	-	0.970 **	0.856 **	0.827 **	0.594 *	0.873 **	0.848 **	0.781 **	0.882 **
b455	0.626 **	0.664 **	0.508 *	0.483	0.595 *	0.873 **	0.970 **	-	0.830 **	0.874 **	0.639 **	0.836 **	0.890 **	0.803 **	0.855 **
b710	0.290	0.280	0.174	0.158	0.785 **	0.821 **	0.856 **	0.830 **	-	0.818 **	0.484	0.672 **	0.777 **	0.737 **	0.970 **
b730	0.604 *	0.487	0.316	0.523 *	0.400	0.911 **	0.827 **	0.874 **	0.818 **	-	0.844 **	0.832 **	0.956 **	0.929 **	0.801 **
d230	0.648 **	0.400	0.249	0.679 **	0.024	0.850 **	0.594 *	0.639 **	0.484	0.844 **	-	0.801 **	0.902 **	0.943 **	0.555 *
d240	0.622 *	0.555 *	0.622 *	0.825 **	0.298	0.926 **	0.873 **	0.836 **	0.672 **	0.832 **	0.801 **	-	0.873 **	0.866 **	0.722 **
d450	0.665 **	0.520 *	0.312	0.555 *	0.397	0.959 **	0.848 **	0.890 **	0.777 **	0.956 **	0.902 **	0.873 **	-	0.983 **	0.832 **
d455	0.611 *	0.416	0.251	0.572 *	0.327	0.963 **	0.781 **	0.803 **	0.737 **	0.929 **	0.943 **	0.866 **	0.983 **	-	0.800 **
s430	0.359	0.320	0.180	0.204	0.775 **	0.875 **	0.882 **	0.855 **	0.970 **	0.801 **	0.555 *	0.722 **	0.832 **	0.800 **	-

FD—Functional domain; b130—Energy and drive functions; b134—Sleep functions; b140—Attention functions; b152—Emotional functions; b280—Sensation of pain; b440—Respiratory functions; b445—Respiratory muscle functions; b455—Exercise tolerance functions; b710—Mobility of joint functions; b730—Muscle power functions; d230—Carrying out daily routine; d240—Handling stress and other psychological demands; d450—Walking; d455—Moving around; s430—Structure of the respiratory system. ** The correlation is significant at *p* < 0.01. * The correlation is significant at *p* < 0.05.

**Table 5:** Impaired function correlations in the long COVID-19 phase.

Long Phase of COVID-19 (r) *p*-Value																	
FD	b130	b134	b140	b152	b280	b440	b445	b455	b710	b730	d230	d240	d450	d455	d850	d920	s430
b130	1	0.895 **	0.533 *	0.528 *	0.401	0.110	−0.197	0.412	0.216	0.526 *	0.781 **	0.653 **	0.384	0.281	0.246	0.688 **	0.252
		0.000	0.041	0.043	0.138	0.696	0.481	0.127	0.439	0.044	0.001	0.008	0.158	0.310	0.442	0.005	0.366
b134	0.895 **	1	0.539 *	0.651 **	0.383	0.217	−0.164	0.270	0.234	0.439	0.824 **	0.788 **	0.439	0.338	0.227	0.640 *	0.351
	0.000		0.038	0.009	0.159	0.437	0.558	0.330	0.401	0.102	0.000	0.000	0.102	0.218	0.478	0.010	0.200
b140	0.533 *	0.539 *	1	0.428	0.711 **	0.403	0.152	0.501	0.535 *	0.704 **	0.675 **	0.410	0.374	0.531 *	0.575	0.699 **	0.564 *
	0.041	0.038		0.111	0.003	0.137	0.587	0.057	0.040	0.003	0.006	0.129	0.169	0.042	0.050	0.004	0.029
b152	0.528 *	0.651 **	0.428	1	0.630 *	0.376	0.000	0.458	0.504	0.455	0.655 **	0.453	0.700 **	0.439	0.246	0.572 *	0.574 *
	0.043	0.009	0.111		0.012	0.167	1.000	0.086	0.055	0.089	0.008	0.090	0.004	0.101	0.442	0.026	0.025
b280	0.401	0.383	0.711 **	0.630 *	1	0.529 *	0.313	0.749 **	0.738 **	0.599 *	0.511	0.303	0.486	0.598 *	0.456	0.559 *	0.666 **
	0.138	0.159	0.003	0.012		0.043	0.256	0.001	0.002	0.018	0.051	0.272	0.066	0.019	0.137	0.030	0.007
b440	0.110	0.217	0.403	0.376	.529 *	1	0.660 **	0.576 *	0.646 **	0.572 *	0.402	0.247	0.718 **	0.835 **	0.625 *	0.606 *	0.809 **
	0.696	0.437	0.137	0.167	0.043		0.007	0.024	0.009	0.026	0.137	0.376	0.003	0.000	0.030	0.017	0.000
b445	−0.197	−0.164	0.152	0.000	0.313	0.660 **	1	0.418	0.745 **	0.433	0.233	−0.066	0.433	0.587 *	0.419	0.267	0.610 *
	0.481	0.558	0.587	1.000	0.256	0.007		0.121	0.001	0.107	0.403	0.816	0.107	0.021	0.176	0.337	0.016
b455	0.412	0.270	0.501	0.458	0.749 **	.576 *	0.418	1	0.681 **	0.690 **	0.474	0.314	0.604 *	0.802 **	0.730 **	0.637 *	0.706 **
	0.127	0.330	0.057	0.086	0.001	0.024	0.121		0.005	0.004	0.074	0.255	0.017	0.000	0.007	0.011	0.003
b710	0.216	0.234	0.535 *	0.504	0.738 **	0.646 **	0.745 **	0.681 **	1	0.760 **	0.573 *	0.115	0.696 **	0.736 **	0.577 *	0.584 *	0.836 **
	0.439	0.401	0.040	0.055	0.002	0.009	0.001	0.005		0.001	0.026	0.683	0.004	0.002	0.049	0.022	0.000
b730	0.526 *	0.439	0.704 **	0.455	0.599 *	0.572 *	0.433	0.690 **	0.760 **	1	0.606 *	0.227	0.792 **	0.799 **	0.453	0.723 **	0.704 **
	0.044	0.102	0.003	0.089	0.018	0.026	0.107	0.004	0.001		0.017	0.415	0.000	0.000	0.140	0.002	0.003
d230	0.781 **	0.824 **	0.675 **	0.655 **	0.511	0.402	0.233	0.474	0.573 *	0.606 *	1	0.673 **	0.538 *	0.489	0.587 *	0.845 **	.622 *
	0.001	0.000	0.006	0.008	0.051	0.137	0.403	0.074	0.026	0.017		0.006	0.038	0.064	0.045	0.000	0.013
d240	0.653 **	0.788 **	0.410	0.453	0.303	0.247	−0.066	0.314	0.115	0.227	0.673 **	1	0.227	0.375	0.289	0.392	0.260
	0.008	0.000	0.129	0.090	0.272	0.376	0.816	0.255	0.683	0.415	0.006		0.415	0.169	0.362	0.149	0.349
d450	0.384	0.439	0.374	0.700 **	0.486	0.718 **	0.433	0.604 *	0.696 **	0.792 **	0.538 *	0.227	1	0.799 **	0.419	0.646 **	0.759 **
	0.158	0.102	0.169	0.004	0.066	0.003	0.107	0.017	0.004	0.000	0.038	0.415		0.000	0.176	0.009	0.001
d455	0.281	0.338	0.531 *	0.439	0.598 *	0.835 **	0.587 *	0.802 **	0.736 **	0.799 **	0.489	0.375	0.799 **	1	0.728 **	0.640 *	0.867 **
	0.310	0.218	0.042	0.101	0.019	0.000	0.021	0.000	0.002	0.000	0.064	0.169	0.000		0.007	0.010	0.000
d850	0.246	0.227	0.575	0.246	0.456	0.625 *	0.419	0.730 **	0.577 *	0.453	0.587 *	0.289	0.419	0.728 **	1	0.756 **	0.896 **
	0.442	0.478	0.050	0.442	0.137	0.030	0.176	0.007	0.049	0.140	0.045	0.362	0.176	0.007		0.004	0.000
d920	0.688 **	0.640 *	0.699 **	0.572 *	0.559 *	0.606 *	0.267	0.637 *	0.584 *	0.723 **	0.845 **	0.392	0.646 **	0.640 *	0.756 **	1	0.760 **
	0.005	0.010	0.004	0.026	0.030	0.017	0.337	0.011	0.022	0.002	0.000	0.149	0.009	0.010	0.004		0.001
S430	0.252	0.351	0.564 *	0.574 *	0.666 **	0.809 **	0.610 *	0.706 **	0.836 **	0.704 **	0.622 *	0.260	0.759 **	0.867 **	0.896 **	0.760 **	1
	0.366	0.200	0.029	0.025	0.007	0.000	0.016	0.003	0.000	0.003	0.013	0.349	0.001	0.000	0.000	0.001	

FD—Functional domain; r—Pearson correlation coefficient; b130—Energy and drive functions; b134—Sleep functions; b140—Attention functions; b152—Emotional functions; b280—Sensation of pain; b440—Respiratory functions; b445—Respiratory muscle functions; b455—Exercise tolerance functions; b710—Mobility of joint functions; b730—Muscle power functions; d230—Carrying out daily routine; d240—Handling stress and other psychological demands; d450—Walking; d455—Moving around; d850—Remunerative employment; d920—Recreation and leisure; s430—Structure of the respiratory system. ** The correlation is significant at *p* < 0.01. * The correlation is significant at *p* < 0.05.

In the long COVID-19 phase, we analyzed the correlation between vaccination status and impairment of the analyzed functions and found a statistically negative correlation. Patients who were not vaccinated showed a strong correlation with impairment of the respiratory structure, paid work, complex movement, joint mobility function, and respiratory functions (Table 6).

### 3.5. Correlations with Vaccination Status

The vaccination status was defined as unvaccinated, fully vaccinated, and partially vaccinated (or incomplete vaccination).

Of the 49 analyzed patients, 19 were unvaccinated, 19 were incompletely vaccinated, and only 11 were vaccinated entirely with three doses. The predominant type of vaccine among the observed groups was Pfizer (76.66%), followed by 16.66% for Astra Zeneca and 6.66% having had mixed vaccines (Astra Zeneca with Pfizer) (Table 7).

In patients with COVID-19 in the first phase of the pandemic, unvaccinated patients (a proportion of 63.2% of patients suffering from COVID-19 in this wave) presented overall impairment of functional domains in more than half of the cases (52.6%).

According to the Romanian authorities’ official releases, there were seven waves of COVID-19 [12].

Wave 1 was characterized by the alpha variant (we did not have any patients from this phase) and lasted until the middle of the year, while the delta variant appeared until the end of 2020 and characterized the second wave. The third and fourth waves had omicron predominance (wave 4 had omicron and delta variants), and waves five, six, and seven had omicron variant infections.

Regarding the analysis based on the pandemic waves, we found one patient with a beta variant infection; most patients had omicron infections (65.3%), with delta variant infections in 17 patients (34.7%). Most vaccinated patients (81.8% with complete vaccination had an omicron variant infection. Most of the unvaccinated patients (73.7%) had delta infections (Table 7).

Long COVID-19 symptoms were found in most patients with omicron infection, followed by delta (Table 8).

The COVID-19 pneumonia was more frequent in the patients with the delta variant, at 88.2%, compared with just 22.60% in patients with omicron variant infection. All patients with delta COVID-19 pneumonia experienced long COVID-19 symptoms, while only 25% of patients with omicron variant pneumonia had long COVID-19.

In the acute phase, the only statistically solid correlation was found between unvaccinated patients and impaired muscle-power function and walking. Moderate association was also highlighted between unvaccinated status and respiratory and respiratory muscle functions, exercise tolerance functions, and handling stress and other psychological demands (Table 9).

In the post-acute phase of COVID-19, we found no significant association between vaccination status and impairments in functional domains (Table 10).

In the long phase of COVID-19, we found several significant correlations between unvaccinated and incomplete vaccination status and impairments in functional domains. The strongest association was determined between unvaccinated and incomplete vaccination status and the structure of the respiratory system, closely followed by moving around and remunerative employment (Table 11).

Long COVID-19 symptoms were found in most patients with omicron infection, followed by delta (Table 11).

The COVID-19 pneumonia was more frequent in the patients with the delta variant, at 88.2%, compared with just 22.60% in patients with omicron variant infection. All patients with delta COVID-19 pneumonia experienced long COVID-19 symptoms, while only 25% of patients with omicron variant pneumonia had long COVID-19 (Table 12 and Table 13).

In the acute phase, energy and impulse functions were closely correlated with sleep functions, daily routine achievement, and coping with stress and other psychological manifestations, compared to the post-acute period. These correlations changed in the post-acute period, being less correlated, although sleep function remained affected. We can conclude that better daily routine management was achieved in the post-acute phase compared to the acute phase. In the long-term COVID-19 phase, energy and impulse functions maintained the strongest correlation with sleep functions, similar to the acute and post-acute phases. Also, the powerful association with carrying out a daily routine reappeared in the long phase of COVID-19, leading to the idea that post-COVID-19 sequelae can affect daily routine more than in the post-acute period (Table 1, Table 4 and Table 5).

Walking is one of the functions that correlates most with respiratory muscle functions in the acute phase and post-acute phase, while the most correlated impairment function in the post-acute phase was complex movement impairment, and correlation was maintained even in the long phase of COVID-19. These functions were equally affected in the acute phase (Table 1, Table 4 and Table 5).

## 4. Discussion

The physiological and pathological mechanisms of COVID-19 involve a complex interplay between the virus, the immune system, and various organs and systems in the body. The virus generates structural changes in the following organs: lung, heart, brain, kidney, blood vessels, liver, muscles and joints, gastrointestinal tract, and skin [13,14,15].

According to the literature, some of these mechanisms are viral entry and replication. The SARS-CoV-2 virus primarily enters the body through the respiratory tract, usually via inhalation of respiratory droplets containing the virus. The virus attaches to receptors on the surface of cells, particularly the angiotensin-converting enzyme 2 (ACE2) receptor, which is abundantly expressed in the respiratory tract but also other tissues such as the heart, kidneys, and gastrointestinal tract. Once inside the cells, the virus replicates, leading to the production of more viral particles (Kumar A, 2021) [16].

Another mechanism involves the immune system, which is critical in combating the virus. Initially, innate immune responses, including the release of pro-inflammatory cytokines and activation of immune cells like macrophages and neutrophils, help to contain the infection. However, in some individuals, an exaggerated immune response can occur, leading to a phenomenon known as a cytokine storm. This dysregulated immune response can cause widespread inflammation and tissue damage, particularly in the lungs and other organs [17].

Lung inflammation and injury in COVID-19 can cause severe pneumonia, characterized by damage to the lung tissue. This can lead to cough, shortness of breath, and respiratory failure, particularly in severe cases. The formation of hyaline membranes and diffuse alveolar damage is often seen in histopathological examinations of lung tissue from COVID-19 patients [18]. COVID-19 is associated with an increased risk of blood clotting and thrombosis, which can lead to complications such as pulmonary embolism, stroke, or heart attack. The virus can directly damage blood vessels and trigger a hypercoagulable condition, which is usually associated with elevated plasma levels of D-dimer, C-reactive protein, P-selectin, and fibrinogen [19].

While COVID-19 primarily affects the respiratory system, it can lead to multi-organ dysfunction [13]. The virus can directly infect other organs, such as the heart, kidneys, liver, and brain, leading to tissue damage and dysfunction. Additionally, systemic inflammation and immune-mediated mechanisms can contribute to organ dysfunction in severe cases [20].

Neurological Effects: COVID-19 can affect the central nervous system, leading to neurological symptoms such as headache, anosmia, ageusia, confusion, and stroke. The exact mechanisms by which the virus affects the nervous system are still being studied but may involve direct viral invasion, inflammation, and immune-mediated damage [21].

Joints and the musculoskeletal system can be affected through inflammatory responses or autoimmune reactions; COVID-19 can trigger a systemic inflammatory response in the body triggered by autoimmune reactions. This inflammatory and autoimmune response can lead to joint pain, swelling, and stiffness and can trigger rheumatoid arthritis or lupus, which are characterized by joint inflammation and damage [22,23]. Severe cases of COVID-19, especially those requiring hospitalization and intensive care, can lead to complications such as blood clots or muscle weakness. These complications may indirectly affect joints and contribute to joint pain or dysfunction [24].

Clinical manifestations found in long-term COVID-19 may result from persistent viral presence or residual viral components. The SARS-CoV-2 virus may persist in the body for an extended period, leading to ongoing inflammation and immune activation. Even if the virus itself is no longer detectable, residual viral components or fragments may continue to trigger immune responses and contribute to symptoms [5]. Long COVID-19 symptoms may also be related to mitochondrial dysfunction, which can impair cellular energy production and contribute to fatigue, muscle weakness, and other symptoms [25].

Based on the ICF model, any change in the body’s structure can also lead to changes in function, which generates limitations in functioning, activities, and participation. The structural modifications during the different phases of COVID-19 led to the functional impairments assessed in this study.

In contrast to the published data, our results showed a solid correlation between sleep functions and pain sensation in the acute phase, followed by impairment of daily routine achievement. The literature describes a bidirectional correlation between sleep deprivation and pain but focuses mainly on chronic pain [26]. As was expected, sleep disorders were prevalent in patients during the acute phase of COVID-19, and many risk factors (older age, anemia, carbon dioxide retention, the number of lobes involved in chest CT, and dyspnea) were identified [26].

In the post-acute phase, sleep functions were significantly correlated only with energy and impulse functions and moderately correlated with attention and effort tolerance functions. In the long COVID-19 phase, sleep impairment also interfered with daily routine achievement and stress adaptability. A moderate correlation can also be observed between sleep functions, emotional functions, and leisure time. Mental functions analyzed in the long COVID-19 phase are analyzed in the temporal context of prolonged symptom persistence and chronic exhaustion of psychological resources, as is also cited in the current literature; respondents frequently nominated stress, depression, and uncertainty regarding COVID-19 as causes of their insomnia [27]. Another study that analyzes sleep dysfunction in post-COVID-19 patients highlights that the prevalence of insomnia increased by 17% after COVID-19 infection, and poor sleep quality was found in more than 40% of patients after 10 months of follow-up. This suggests that neuroinflammation is the first involved response to transient neuronal damage due to a SARS-CoV-2-induced pro-inflammatory state, and then psychological disorders, particularly anxiety [28]. In a similar study of 501 persons, the most commonly impaired mental functions reported by participants were sleep problems (71.9%), stress (73%), problems in organization and planning (73.9%), language problems (80.6%), memory problems (84.3%), concentration problems (84.5%), and attention problems (84.7%) [29].

In the acute phase, attention functions presented a weak correlation with any other functions, being only moderately correlated with sleep, energy and impulse functions, and emotional functions. In the literature, there are no objective studies that evaluated attention in the acute phase, but there are data that highlight that post-COVID-19 patients show lower performance across multiple tasks such as tapping attention, executive functions, and language [30]. In our study, in patients in a chronic phase of COVID-19, we found a robust statistical correlation between attention and emotional functions, followed by, surprisingly, the association between attention function impairment and the sensation of pain. A direct causal relationship cannot be deduced, but it must be taken into account that patients who reach the long-term COVID-19 phase with symptoms suffer from chronic pain and are also administered medication in this regard, which can cause adverse reactions. This was not the subject of our research. Moreover, there are no relevant publications on this topic. The only function found in our study that had a feeble correlation with attention was carrying out daily routine activities in the acute phase of the infection, with moderate to strong correlations between this and all other analyzed functions.

Regarding emotional functions, when affected, the impact was weak or moderate on all functions, but in the acute phase, the strongest correlation was established between these and respiratory muscle functions. Although causality cannot be determined according to the ClinFIT-COVID-19 Manual, the dysfunction of respiratory muscles includes paradoxical movements of the abdominal musculature or alternating respiratory movements, causing respiratory stress, which can influence emotional functions. In the post-acute phase, a close relationship was established between emotional functions and stress adaptation. A previously published study that evaluated cognitive deficits and emotional distress among COVID-19 and post-COVID-19 patients who required functional rehabilitation concluded that extended neuropsychological dysfunctions are found in patients who recovered in functional rehabilitation units for COVID-19 (in the subacute phase of the disease) or post-COVID-19 patients [31]. During the long COVID-19 phase, a statistically significant correlation was surprisingly found between emotional functions and walking impairment. Walking is generally one of the functions that contributes the most to an individual’s functional independence. Although the literature does not correlate these two aspects in COVID-19 patients, our results showed a significant relationship.

**Figure 6:**
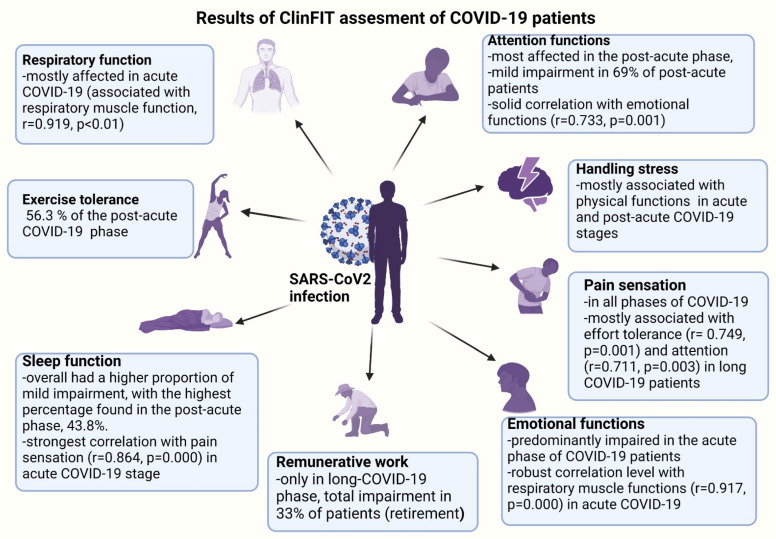
Results of ClinFIT tool evaluation of patients in different stages of COVID-19 infection.

The pain sensation was closely related to the impairment of daily routine and sleep functions and moderately correlated with all other analyzed functions in the acute phase. In the post-acute phase, a decreased coefficient of association between the above-mentioned functions was noted (Figure 5 and Figure 6).

Similar to the literature reports, our study found a good statistical correlation between pain and mobility functions and the structure of the respiratory system [32]. Over time, in the long COVID-19 phase, our results showed that the correlation level between pain and mobility was maintained, with the appearance of a close association with effort tolerance and attention. Although few published studies correlate pain sensation with other domain impairments, there is scarce data regarding pain in the long COVID-19 context [32]. The data so far have shown that myalgia, headache, and chest pain can be seen in patients at varying rates; myalgia and headache, especially, are among the initial symptoms [33]. Additionally, COVID-19 survivors can experience chronic complications as a consequence of prolonged hospital stays, immobility, long-term mechanical ventilation, and other therapies. Pain conditions, mostly expressed as myalgia or joint pain, can frequently be found among these COVID-19 sequelae [34].

Respiratory and respiratory muscle functions were close to moderately correlated with functions related to physical activity, and they had a weaker correlation with mental functions.

In the post-acute phase, the association between respiratory functions, respiratory muscle functions, and the emotional component is weakened, and significant statistical associations with other physical functions of the body were found. In the long COVID-19 phase, when pulmonary inflammation usually decreases but pulmonary sequels are installed, respiratory muscle function is no longer closely correlated with respiratory muscle functions and effort tolerance [35]. Because COVID-19 is a virus with pulmonary tropism, respiratory function was one of the most studied areas. Several studies conclude that post-infection COVID-19 patients showed impaired lung function; the most important of the pulmonary function tests affected was the diffusion capacity [36].

In the acute phase, joint mobility functions were most closely correlated with respiratory functions and only moderately with walking because, in the acute phase, patients are in isolation without needing to travel long distances. A study that highlighted the link between COVID-19 and respiratory muscle functions concluded that COVID-19 impacts the respiratory muscles, particularly the diaphragm, by forcing mechanical ventilation and the consequent muscle inactivity in ICU conditions and through mechanisms linked with SARS-CoV-2 viral infection [34].

In the post-acute phase, the impairment of joint mobility was most closely correlated with the structure of the respiratory system, followed by respiratory muscle functions and effort tolerance, with which a high statistical association is maintained. A study that evaluated symptoms in the post-acute phase of COVID-19 concluded that musculoskeletal symptoms (joint stiffness, joint pain, and myalgia) are among the most prevalent and persistent symptoms in people with post-COVID-19 conditions [37].

The most important statistical association was also maintained in the long COVID-19 phase with the structure of the respiratory system but was slightly lower than in the post-acute phase. This was followed by the association with impairment of muscle strength function and, to a similar extent, with respiratory functions, moving around, and pain sensation.

According to a single-center experience study, reporting on 1200 subjects, cryptogenic organizing pneumonia, ground glass opacities, and fibrosis were common post-COVID-19 sequelae [38].

Similar to respiratory and respiratory muscle functions, muscle strength function was closely correlated with functions based on physical activity and moderately correlated with other functions in the acute phase and with statistically more significant importance in the post-acute phase. In the long-term COVID-19 phase, when pulmonary inflammation usually decreases, muscle strength function is no longer closely correlated with respiratory functions, respiratory muscles, and effort tolerance.

Additionally, muscle strength functions are not correlated in the long COVID-19 phase with paid work impairment as much as they are associated with the structure of the respiratory system, impairment of complex movement, and effort tolerance. In a study, it was demonstrated that patients with long COVID-19 syndrome have lower absolute and relative muscle strength than control participants, and this relationship may be mediated mainly by appendicular lean mass index [39].

In the acute and long COVID-19 phases, daily routine achievement was most closely correlated with mental functions (sleep, energy and impulse, attention), while in the post-acute phase, impairment of daily routine achievement was most closely correlated with walking, complex movement, and muscle power functions. A robust meta-analysis concluded the positive association between daily routine disruptions and symptoms of mental disorders among large populations that suffered from COVID-19 across different geo-temporal contexts [40].

Handling stress and other psychological manifestations were most closely correlated with muscle power functions, walking, and energy and impulse function in the acute phase, followed in the post-acute phase by being most closely correlated with respiratory functions, respiratory muscles, walking, and complex movement. This pattern does not persist in the long COVID-19 phase, where stress adaptation is intensely associated only with sleep function impairment. The cause of persisting symptoms may be linked to the direct action of the coronavirus on the brain and CNS, indirect effects via systemic inflammatory responses to the virus, or a result of psychological stressors such as being infected, stigma, and the experience of being in the ICU, according to Thye et al. [41]. Also, they showed that COVID-19’s mental health consequences continue even after hospital discharge [41].

In the acute phase, walking was correlated with all functions, mostly with a close and moderate correlation, being most intensely correlated with respiratory muscle dysfunction. In the post-acute phase, when the patient usually re-enters the community, locomotion dysfunction was most intensely associated with muscle weakness, respiratory dysfunction, and complex movement impairment, a correlation that persists in the long COVID-19 phase. A study that analyzed walking during and after COVID-19 infections demonstrated that individuals in the infected by COVID-19 group presented changes in the ranges of motion and the time to complete the timed-up and go test task, even though at least eight weeks passed after hospital discharge [10].

Moving around was assessed only in the post-acute and long COVID-19 stages when the patient exited the infectious phase and had the opportunity to move in various conditions. In these two phases, there were similar close correlations between complex movements.

The impairment of remunerative employment was analyzed only in the long COVID-19 phase, a temporal moment when the patient typically returns to their usual life schedule, and it was strongly associated with the impairment of the respiratory system structure and similar effort intolerance, to a similar extent as muscle weakness. There were patients with complete impairment of this function, finding themselves in a situation of interrupting paid work performance. Patients who experienced impairment in recreation and leisure activities typically also showed a close correlation with impairment in carrying out their daily routine, impairment of the respiratory system structure, paid work, and muscle strength function. Additionally, musculoskeletal symptoms might be the most clinically significant manifestation of the post-COVID-19 condition for patients and their caregivers because they limit physical and functional performance and impede return to work [40]. Also, cognitive decline, chronic fatigue, and mental health deterioration are symptoms that can create challenges for individuals seeking to return to their professional roles. Cognitive impairments, such as difficulties with concentration and memory, can impact job performance and decision-making abilities [42].

The structure of the respiratory system was analyzed in the post-acute and long COVID-19 phases after the decrease in the cytokine storm at the alveolar level, finding that there is no close association between the impairment of the respiratory system structure and the achievement of the usual daily schedule; as expected, the correlation was moderate. In the post-acute phase, the structure of the respiratory system was most closely associated with impairment of joint mobility while maintaining statistically significant associations with other physical functions of the body. In the long-term COVID-19 phase, the same close statistical associations are kept, except for the moderate association with respiratory muscle functions, and there remains a weak association between the structure of the respiratory system and adaptation to stress and other psychological manifestations. Further studies have found a correlation between the pulmonary CT appearance in patients with moderate impairment and “respiratory functions” and between the CT appearance of the lungs and “emotional functions”, especially in patients in the long COVID-19 phase [43].

In the survey, most patients experiencing long COVID-19 symptoms were infected with the delta variant during the first year of COVID-19 in Romania and with the omicron variant in subsequent waves. Our analysis indicates that the delta variant was generally associated with more severe impairments across all functional domains than the omicron variant. The most pronounced difference was observed in emotional functions, potentially attributable to the widespread psychological impact during the early phase of the pandemic, as reported in a systematic review of the impact of the first wave of COVID-19 on psychological and psychosocial dimensions [44].

Additionally, our study found that muscle power functions and complex movements were moderately to severely affected in patients infected with the delta variant, whereas those infected with the omicron variant exhibited only mild impairments. A notable difference was also observed in the structure of the respiratory system; delta variant patients experienced more significant impairments than the predominantly mild impairments in omicron variant patients. We also identified an association between the delta variant and recreation impairment, which correlated with more severe mobility limitations, potentially leading to functional impairments in this domain.

The literature supports that delta variant infection tends to result in more severe symptoms, as demonstrated in a study involving nearly 150,000 patients [45]. Notably, only patients infected with the delta variant experienced complete impairment in remunerated work, indicating a more severe functional impact.

Our study’s limitations were mainly related to the limited number of participants for each phase, of which only 11 patients included in the study were fully vaccinated. Further research with larger, more diverse cohorts is needed to confirm the findings. However, we highlight that the strength of our research is the complex assessment using an innovative and comprehensive tool such as ClinFIT-COVID-19, which covers a wide range of functioning aspects, including body functions and structures, activities, participation, and environmental factors. This allowed the study to capture the full impact of COVID-19 on patients’ lives, from physical impairments to participation restrictions in daily life.

By statistically analyzing these data, the variability of manifestations of impairment of function and structure of the body in patients from Romania collected multicentrically in various stages of SARS-CoV-2 infection was highlighted. This analysis leads to the idea of a personalized rehabilitation plan within a multidisciplinary team, aimed at improving individual functioning in society based on structural remains. Since a significant percentage of 33.3% of long COVID-19 patients who were employed retired after COVID-19, the issue of implementing a professional recovery evaluation arises, and this evaluation should be part of the rehabilitation plan.

## 5. Conclusions

By identifying specific areas of impairment and activity limitation, the study can guide the development of tailored rehabilitation programs and interventions, enhancing their effectiveness. Since the ICF is internationally recognized, the study’s findings can be compared with other studies globally, improving the understanding of post-COVID-19 functioning across different populations and healthcare settings. Using a standardized classification system allows for benchmarking against best practices and helps identify successful strategies that can be replicated or adapted in other contexts. By leveraging the ICF classification, the study on functioning in post-COVID-19 patients can offer a nuanced, comprehensive, and actionable understanding of the challenges faced by these individuals, ultimately contributing to improved health outcomes and quality of life.

## Figures and Tables

**Table 1:** Impaired function correlations in acute COVID-19 patients.

Pearson Correlation Coefficient		Acute Phase of COVID-19												
		b130	b134	b140	b152	b280	b440	b445	b455	b710	b730	d230	d240	d450
b130	r	1	0.733 **	0.498 *	0.650 **	0.584 *	0.292	0.449	0.260	0.041	0.580 *	0.763 **	0.775 **	0.589 *
	*p*-value		0.001	0.035	0.004	0.011	0.239	0.062	0.298	0.873	0.012	0.000	0.000	0.010
b134	r	0	1	0.461	0.446	0.864 **	0.433	0.458	0.384	0.385	0.558 *	0.804 **	0.569 *	0.594 **
	*p*-value	0.001		0.054	0.063	0.000	0.073	0.056	0.116	0.114	0.016	0.000	0.014	0.009
b140	r	0.498 *	0.461	1	0.447	0.301	0.264	0.373	0.000	0.059	0.325	0.283	0.269	0.301
	*p*-value	0.035	0.054		0.063	0.224	0.290	0.128	1.000	0.817	0.188	0.255	0.281	0.224
b152	r	0.650 **	0.446	0.447	1	0.365	0.763 **	0.917 **	0.724 **	0.416	0.849 **	0.632 **	0.768 **	0.899 **
	*p*-value	0.004	0.063	0.063		0.136	0.000	0.000	0.001	0.086	0.000	0.005	0.000	0.000
b280	r	0.584 *	0.864 **	0.301	0.365	1	0.412	0.431	0.457	0.411	0.559 *	0.710 **	0.555 *	0.533 *
	*p*-value	0.011	0.000	0.224	0.136		0.090	0.074	0.056	0.090	0.016	0.001	0.017	0.023
b440	r	0.292	0.433	0.264	0.763 **	0.412	1	0.919 **	0.858 **	0.852 **	0.847 **	0.622 **	0.506 *	0.882 **
	*p*-value	0.239	0.073	0.290	0.000	0.090		0.000	0.000	0.000	0.000	0.006	0.032	0.000
b445	r	0.449	0.458	0.373	0.917 **	0.431	0.919 **	1	0.816 **	0.686 **	0.889 **	0.685 **	0.624 **	0.917 **
	*p*-value	0.062	0.056	0.128	0.000	0.074	0.000		0.000	0.002	0.000	0.002	0.006	0.000
b455	r	0.260	0.384	0.000	0.724 **	0.457	0.858 **	0.816 **	1	0.724 **	0.848 **	0.505 *	0.667 **	0.855 **
	*p*-value	0.298	0.116	1.000	0.001	0.056	0.000	0.000		0.001	0.000	0.032	0.002	0.000
b710	r	0.041	0.385	0.059	0.416	0.411	0.852 **	0.686 **	0.724 **	1	0.707 **	0.513 *	0.240	0.652 **
	*p*-value	0.873	0.114	0.817	0.086	0.090	0.000	0.002	0.001		0.001	0.030	0.337	0.003
b730	r	0.580 *	0.558 *	0.325	0.849 **	0.559 *	0.847 **	0.889 **	0.848 **	0.707 **	1	0.709 **	0.818 **	0.913 **
	*p*-value	0.012	0.016	0.188	0.000	0.016	0.000	0.000	0.000	0.001		0.001	0.000	0.000
d230	r	0.763 **	0.804 **	0.283	0.632 **	0.710 **	0.622 **	0.685 **	0.505 *	0.513 *	0.709 **	1	0.613 **	0.728 **
	*p*-value	0.000	0.000	0.255	0.005	0.001	0.006	0.002	0.032	0.030	0.001		0.007	0.001
d240	r	0.775 **	0.569 *	0.269	0.768 **	0.555 *	0.506 *	0.624 **	0.667 **	0.240	0.818 **	0.613 **	1	0.796 **
	*p*-value	0.000	0.014	0.281	0.000	0.017	0.032	0.006	0.002	0.337	0.000	0.007		0.000
d450	r	0.589 *	0.594 **	0.301	0.899 **	0.533 *	0.882 **	0.917 **	0.855 **	0.652 **	0.913 **	0.728 **	0.796 **	1
	*p*-value	0.010	0.009	0.224	0.000	0.023	0.000	0.000	0.000	0.003	0.000	0.001	0.000	

** The correlation is significant at *p* < 0.01. * The correlation is significant at *p* < 0.05. r—Pearson correlation coefficient; b130—Energy and drive functions; b134—Sleep functions; b140—Attention functions; b152—Emotional functions; b280—Sensation of pain; b440—Respiratory functions; b445—Respiratory muscle functions; b455—Exercise tolerance functions; b710—Mobility of joint functions; b730—Muscle power functions; d230—Carrying out daily routine; d240—Handling stress and other psychological demands; d450—Walking.

**Table 2:** Need of assistance, assistive devices, and oxygen in the COVID-19 acute context.

COVID-19 Acute Phase	The Pearson Correlation Coefficient			
Functional Domain (FD)	Age (r)	O_2_ Necessary (r)	Assistance Need (r)	Assistive Devices Need (r)
b130	−0.298	0.230	0.230	0.509 *
b134	0.090	0.486 *	0.486 *	0.587 *
b140	−0.430	0.158	0.158	0.538 *
b152	−0.270	0.530 *	0.530 *	0.735 **
b280	−0.030	0.397	0.397	0.525 *
b440	0.055	0.870 **	0.870 **	0.736 **
b445	−0.092	0.707 **	0.707 **	0.713 **
b455	0.133	0.706 **	0.706 **	0.654 **
b710	0.336	0.867 **	0.867 **	0.550 *
b730	−0.093	0.686 **	0.686 **	0.713 **
d230	0.075	0.559 *	0.559 *	0.465
d240	−0.230	0.378	0.378	0.607 **
d450	−0.014	0.794 **	0.794 **	0.826 **

** The correlation is significant at *p* < 0.01. * The correlation is significant at *p* < 0.05. r—Pearson correlation coefficient; b130—Energy and drive functions; b134—Sleep functions; b140—Attention functions; b152—Emotional functions; b280—Sensation of pain; b440—Respiratory functions; b445—Respiratory muscle functions; b455—Exercise tolerance functions; b710—Mobility of joint functions; b730—Muscle power functions; d230—Carrying out daily routine; d240—Handling stress and other psychological demands; d450—Walking.

**Table 3:** Need of assistance, assistive devices, and oxygen in the post-acute COVID-19 context.

Post-Acute COVID-19 Phase	Pearson Correlation Coefficient			
	Age (r)	O_2_ Necessary (r)	Assistance Need (r)	Assistive Devices Need (r)
b130	0.478	0.460	0.460	0.460
b134	0.054	0.194	0.194	0.194
b140	−0.304	0.121	0.121	0.121
b152	0.049	0.550 *	0.550 *	0.550 *
b280	0.298	0.174	0.174	0.174
b440	0.564 *	0.885 **	0.885 **	0.885 **
b445	0.382	0.595 *	0.595 *	0.595 *
b455	0.430	0.577 *	0.577 *	0.577 *
b710	0.515 *	0.602 *	0.602 *	0.602 *
b730	0.559 *	0.799 **	0.799 **	0.799 **
d230	0.575 *	0.935 **	0.935 **	0.935 **
d240	0.366	0.778 **	0.778 **	0.778 **
d450	0.619 *	0.866 **	0.866 **	0.866 **

** The correlation is significant at *p* < 0.01. * The correlation is significant at *p* < 0.05. r—Pearson correlation coefficient; b130—Energy and drive functions; b134—Sleep functions; b140—Attention functions; b152—Emotional functions; b280—Sensation of pain; b440—Respiratory functions; b445—Respiratory muscle functions; b455—Exercise tolerance functions; b710—Mobility of joint functions; b730—Muscle power functions; d230—Carrying out daily routine; d240—Handling stress and other psychological demands; d450—Walking.

**Table 6:** Need of assistance, assistive devices, and oxygen, in the long-term COVID-19 context.

Long- Term COVID-19	Pearson Correlation Coefficient				
Functional Domain (FD)	Age (r)	O_2_ Necessary (r)	Assistance Need (r)	Assistive Devices Need (r)	Vaccinated/NO (r)
b130	0.157	0.171	0.546 *	0.355	−0.009
b134	0.346	0.261	0.587 *	0.125	−0.124
b140	0.565 *	0.484	0.601 *	0.233	−0.447
b152	0.442	0.393	0.653 **	0.248	−0.290
b280	0.374	0.543 *	0.610 *	0.315	−0.363
b440	0.476	0.645 **	0.639 *	0.521 *	−0.739 **
b445	0.452	0.577 *	0.426	0.277	−0.661 **
b455	0.296	0.690 **	0.637 *	0.663 **	−0.565 *
b710	0.547 *	0.760 **	0.795 **	0.426	−0.704 **
b730	0.355	0.583 *	0.892 **	0.681 **	−0.600 *
d230	0.649 **	0.538 *	0.696 **	0.259	−0.485
d240	0.275	0.227	0.308	−0.073	−0.199
d450	0.430	0.583 *	0.892 **	0.681 **	−0.600 *
D455	0.420	0.799 **	0.814 **	0.617 *	−0.817 **
D850	0.548	0.942 **	0.596 *	0.596 *	−0.825 **
D920	0.573 *	0.646 **	0.761 **	0.606 *	−0.614 *
s430	0.591 *	0.924 **	0.845 **	0.550 *	−0.879 **

r—Pearson correlation coefficient; b130—Energy and drive functions; b134—Sleep functions; b140—Attention functions; b152—Emotional functions; b280—Sensation of pain; b440—Respiratory functions; b445—Respiratory muscle functions; b455—Exercise tolerance functions; b710—Mobility of joint functions; b730—Muscle power functions; d230—Carrying out daily routine; d240—Handling stress and other psychological demands; d450—Walking; d455—Moving around; d850—Remunerative employment; d920—Recreation and leisure; s430—Structure of the respiratory system. ** The correlation is significant at *p* < 0.01 level. * The correlation is significant at *p* < 0.05.

**Table 7:** Vaccination status and type of virus variant distribution in the studied cohort.

		Total							
		Total		Unvaccinated (0 Doses)		Incomplete (1–2 Doses)		Complete (3 Doses)	
		Count	%	Count	%	Count	%	Count	%
Vaccine type	Total	49	49	19	19	19	19	11	11
	Unvaccinated	19	38.8%	19	100.0%	0	0.0%	0	0.0%
	Pfizer	23	46.9%	0	0.0%	14	73.7%	9	81.8%
	Astra Zeneca	5	10.2%	0	0.0%	5	26.3%	0	0.0%
	2 Astra Zeneca, 1 Pfizer	2	4.1%	0	0.0%	0	0.0%	2	18.2%
COVID-19 Variant	Total	49	49	19	19	19	19	11	11
	Beta	1	2.0%	0	0.0%	0	0.0%	1	9.1%
	Omicron	32	65.3%	6	31.6%	17	89.5%	9	81.8%
	Delta	17	34.7%	14	73.7%	2	10.5%	1	9.1%
Wave	Total	49	49	19	19	19	19	11	11
	Waves 1 + 2	13	26.5%	12	63.2%	1	5.3%	0	0.0%
	Waves 3 + 4	6	12.2%	3	15.8%	2	10.5%	1	9.1%
	Waves 5 + 6	8	16.3%	2	10.5%	5	26.3%	1	9.1%
	Waves 7	23	46.9%	3	15.8%	11	57.9%	9	81.8%

**Table 8:** Distribution of COVID-19 pneumonia and hospitalization in studied cohort.

		Total		Acute		Post-Acute		Long COVID-19	
		Count	%	Count	%	Count	%	Count	%
Vaccine type	Total	49	49	18	18	16	16	15	15
	Unvaccinated	19	38.8%	8	44.4%	4	25.0%	7	46.7%
	Pfizer	23	46.9%	9	50.0%	8	50.0%	6	40.0%
	Astra Zeneca	5	10.2%	0	0.0%	3	18.8%	2	13.3%
	2 Astra Zeneca, 1 Pfizer	2	4.1%	1	5.6%	1	6.3%	0	0.0%
COVID-19 variant	Total	49	49	18	18	16	16	15	15
	Beta	1	2.0%	1	5.6%	0	0.0%	0	0.0%
	Omicron	32	65.3%	12	66.7%	11	68.8%	9	60.0%
	Delta	17	34.7%	5	27.8%	5	31.3%	7	46.7%
COVID-19 pneumonia		49	49	18	18	16	16	15	15
	No	24	49.0%	10	55.6%	8	50.0%	6	40.0%
	Yes	25	51.0%	8	44.4%	8	50.0%	9	60.0%
Hospitalization		49	49	18	18	16	16	15	15
	No	26	53.1%	10	55.6%	11	68.8%	5	33.3%
	Yes	23	46.9%	8	44.4%	5	31.3%	10	66.7%
Wave	Total	49	49	18	18	16	16	15	15
	Waves 1 + 2	13	26.5%	5	27.8%	3	18.8%	5	33.3%
	Waves 3 + 4	6	12.2%	0	0.0%	2	12.5%	4	26.7%
	Waves 5 + 6	8	16.3%	4	22.2%	1	6.3%	3	20.0%
	Waves 7	23	46.9%	9	50.0%	10	62.5%	4	26.7%

**Table 9:** Correlation between vaccination status and functional domains in the acute phase of COVID-19.

Correlations—Acute Phase of COVID-19	Age	O_2_ Necessary	Assistance Need	Assistive Devices Need	Complete Vaccination	Incomplete Vaccination	Unvaccinated
b130 Energy and drive functions	−0.298	0.230	0.230	0.509 *	−0.050	−0.263	0.291
b134 Sleep functions	0.090	0.486 *	0.486 *	0.587 *	−0.073	−0.388	0.430
b140 Attention functions	−0.430	0.158	0.158	0.538 *	0.359	−0.474 *	0.150
b152 Emotional functions	−0.270	0.530 *	0.530 *	0.735 **	−0.267	−0.353	0.559 *
b280 Sensation of pain	−0.030	0.397	0.397	0.525 *	−0.285	−0.318	0.540 *
b440 Respiratory functions	0.055	0.870 **	0.870 **	0.736 **	−0.447	−0.278	0.638 **
b445 Respiratory muscle functions	−0.092	0.707 **	0.707 **	0.713 **	−0.356	−0.353	0.634 **
b455 Exercise tolerance functions	0.133	0.706 **	0.706 **	0.654 **	−0.427	−0.353	0.692 **
b710 Mobility of joint functions	0.336	0.867 **	0.867 **	0.550 *	−0.398	−0.247	0.568 *
b730 Muscle power functions	−0.093	0.686 **	0.686 **	0.713 **	−0.308	−0.472 *	0.705 **
d230 Carrying out daily routine	0.075	0.559 *	0.559 *	0.464	−0.296	−0.223	0.460
d240 Handling stress and other psychological demands	−0.230	0.378	0.378	0.607 **	−0.196	−0.472 *	0.613 **
d450 Walking	−0.014	0.794 **	0.794 **	0.826 **	−0.390	−0.397	0.703 **

** The correlation is significant at *p* < 0.01. * The correlation is significant at *p* < 0.05.

**Table 10:** Correlation between vaccination status and functional domains in the post-acute phase of COVID-19.

Correlations—Post-Acute Phase of COVID-19	Age	O_2_ Necessary	Assistance Need	Assistive devices Need	Complete Vaccination	Incomplete Vaccination	Unvaccinated
b130 Energy and drive functions	0.478	0.460	0.460	0.460	−0.510 *	0.417	0.104
b134 Sleep functions	0.054	0.194	0.194	0.194	−0.372	0.289	0.092
b140 Attention functions	−0.304	0.121	0.121	0.121	0.139	−0.046	−0.104
b152 Emotional functions	0.049	0.550 *	0.550 *	0.550 *	−0.211	0.211	0.000
b280 Sensation of pain	0.298	0.174	0.174	0.174	−0.333	0.022	0.348
b440 Respiratory functions	0.564 *	0.885 **	0.885 **	0.885 **	−0.490	0.264	0.253
b445 Respiratory muscle functions	0.382	0.595 *	0.595 *	0.595 *	−0.358	0.162	0.218
b455 Exercise tolerance functions	0.430	0.577 *	0.577 *	0.577 *	−0.425	0.254	0.190
b710 Mobility of joint functions	0.515 *	0.602 *	0.602 *	0.602 *	−0.484	0.317	0.187
b730 Muscle power functions	0.559 *	0.799 **	0.799 **	0.799 **	−0.537 *	0.454	0.092
d230 Carrying out daily routine	0.575 *	0.935 **	0.935 **	0.935 **	−0.501 *	0.358	0.160
d240 Handling stress and other psychological demands	0.366	0.778 **	0.778 **	0.778 **	−0.447	0.298	0.167
d450 Walking	0.619 *	0.866 **	0.866 **	0.866 **	−0.566 *	0.370	0.218
d455 Moving around (G)	0.644 **	0.944 **	0.944 **	0.944 **	−0.568 *	0.361	0.231
s430 Structure of the respiratory system	0.573 *	0.674 **	0.674 **	0.674 **	−0.516 *	0.258	0.289

** The correlation is significant at *p* < 0.01. * The correlation is significant at *p* < 0.05.

**Table 11:** Correlation between vaccination status and functional domains in the phase of long COVID-19.

Correlations—Long Phase of COVID-19	Age	O_2_ Necessary	Assistance Need	Assistive Need	Complete Vaccination	Incomplete Vaccination	Unvaccinated
b130 Energy and drive functions	0.157	0.171	0.546 *	0.355	−0.037	0.009	0.009
b134 Sleep functions	0.346	0.261	0.587 *	0.125	−0.031	−0.109	0.124
b140 Attention functions	0.565 *	0.484	0.601 *	0.233	−0.058	−0.418	0.447
b152 Emotional functions	0.442	0.393	0.653 **	0.248	−0.193	−0.193	0.290
b280 Sensation of pain	0.374	0.543 *	0.610 *	0.315	0.104	−0.414	0.363
b440 Respiratory functions	0.476	0.645 **	0.639 *	0.521 *	0.211	−0.844 **	0.739 **
b445 Respiratory muscle functions	0.452	0.577 *	0.426	0.277	0.189	−0.756 **	0.661 **
b455 Exercise tolerance functions	0.296	0.690 **	0.637 *	0.663 **	−0.226	−0.451	0.565 *
b710 Mobility of joint functions	0.547 *	0.760 **	0.795 **	0.426	−0.083	−0.663 **	0.704 **
b730 Muscle power functions	0.355	0.583 *	0.892 **	0.681 **	−0.218	−0.490	0.600 *
d230 Carrying out daily routine	0.649 **	0.538 *	0.696 **	0.259	−0.088	−0.440	0.485
d240 Handling stress and other psychological demands	0.275	0.227	0.308	−0.073	−0.050	−0.174	0.199
d450 Walking	0.430	0.583 *	0.892 **	0.681 **	−0.218	−0.490	0.600 *
d455 Moving around (G)	0.422	0.799 **	0.814 **	0.617 *	−0.174	−0.730 **	0.817 **
d850 Remunerative employment	0.548	0.942 **	0.596 *	0.596 *	−0.134	−0.740 **	0.825 **
d920 Recreation and leisure	0.573 *	0.646 **	0.761 **	0.606 *	−0.040	−0.594 *	0.614 *
s430 Structure of the respiratory system	0.591 *	0.924 **	0.845 **	0.550 *	−0.058	−0.850 **	0.879 **

** The correlation is significant at *p* < 0.01. * The correlation is significant at *p* < 0.05.

**Table 12:** Overall hospitalization for COVID-19.

		Total		Acute Phase		Post-Acute Phase		Long COVID-19	
		Count	%	Count	%	Count	%	Count	%
Hospitalization		49	49	18	18	16	16	15	15
	No	26	53.1%	10	55.6%	11	68.8%	5	33.3%
	Yes	23	46.9%	8	44.4%	5	31.3%	10	66.7%

**Table 13:** Correlation with COVID-19 and hospitalization in the studied cohort.

Correlations	Hospitalization			
	Total	Acute-Phase	Post-Acute Phase	Long COVID-19
COVID-19 pneumonia	0.758 **	0.775 **	0.674 **	0.866 **
b130 Energy and drive functions	0.299 *	0.291	0.460	0.246
b134 Sleep functions	0.323 *	0.568 *	0.194	0.329
b140 Attention functions	0.175	0.000	0.121	0.381
b152 Emotional functions	0.528 **	0.559 *	0.550 *	0.511
b280 Sensation of pain	0.489 **	0.653 **	0.174	0.431
b440 Respiratory functions	0.799 **	0.836 **	0.885 **	0.660 **
b445 Respiratory muscle functions	0.594 **	0.745 **	0.595 *	0.400
b455 Exercise tolerance functions	0.688 **	0.893 **	0.577 *	0.418
B710 Mobility of joint functions	0.671 **	0.833 **	0.602 *	0.482
B730 Muscle power functions	0.603 **	0.827 **	0.799 **	0.289
D230 Carrying out daily routine	0.743 **	0.672 **	0.935 **	0.653 **
D240 Handling stress and other psychological demands	0.570 **	0.613 **	0.778 **	0.328
D450 Walking	0.662 **	0.816 **	0.866 **	0.433
D455 Moving around	0.730 **	.^c^	0.944 **	0.462
D850 Remunerative employment	0.732 **	.^c^	.^c^	0.732 **
D920 Recreation and leisure	0.746 **	.^c^	.^c^	0.746 **
S430 Structure of the respiratory system	0.700 **	.^c^	0.674 **	0.724 **
Vaccination status	−0.601 **	−0.743 **	−0.453	−0.463
O_2_ necessary	0.775 **	0.791 **	1.000 **	0.577 *
Need of assistance	0.706 **	0.791 **	1.000 **	0.426
Need of assistive devices	0.572 **	0.598 **	1.000 **	0.277

** The correlation is significant at *p* < 0.01; * The correlation is significant at *p* < 0.05; .^c^—missing data.

## Data Availability

Data is available upon reasonable request.
